# Supplementary material for: FHL1 promotes chikungunya and o’nyong-nyong virus infection and pathogenesis with implications for alphavirus vaccine design
Source: Nat Commun. 2023 Oct 26;14:6605. doi: 10.1038/s41467-023-42330-2 (PMC10603155; doi:10.1038/s41467-023-42330-2)

Supplementary Fig S1

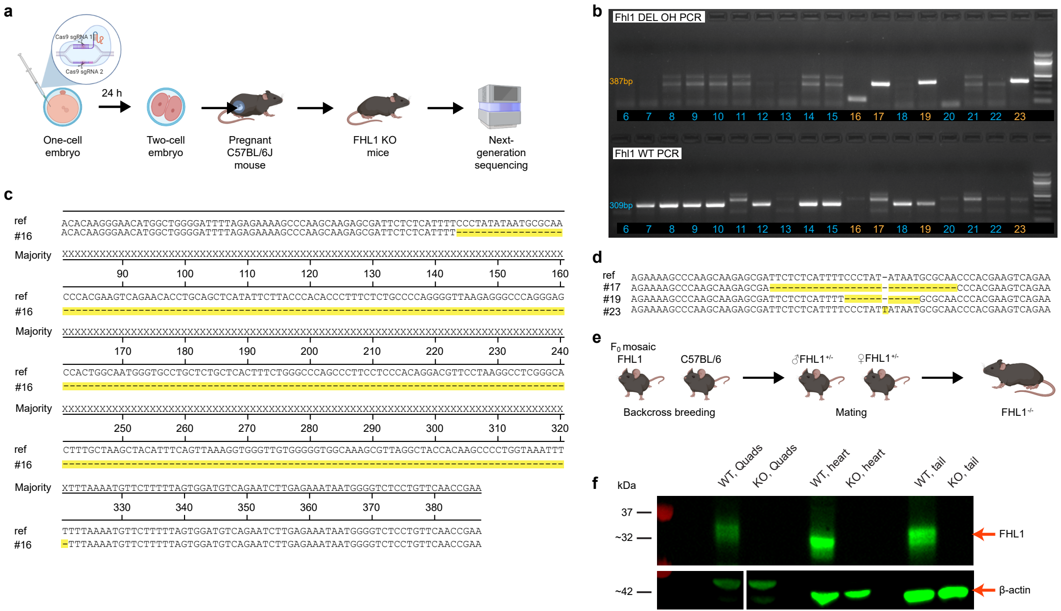

Supplementary Fig S2

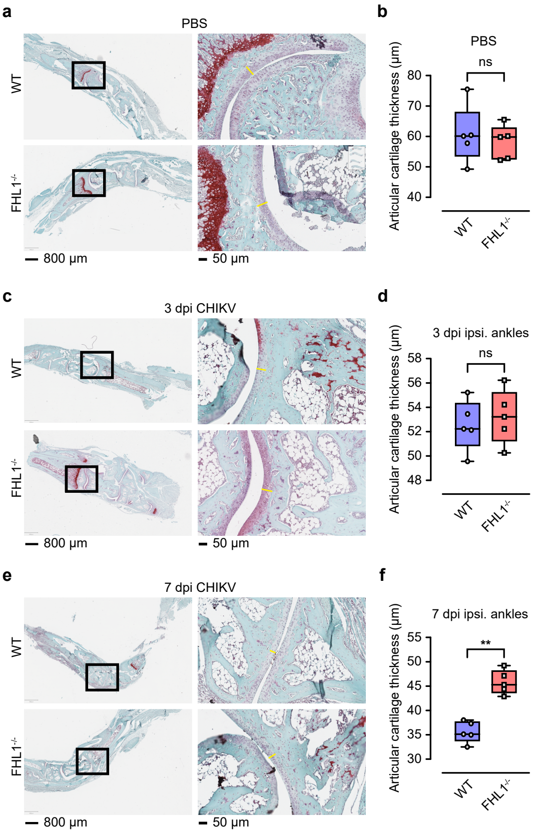

Supplementary Fig S3

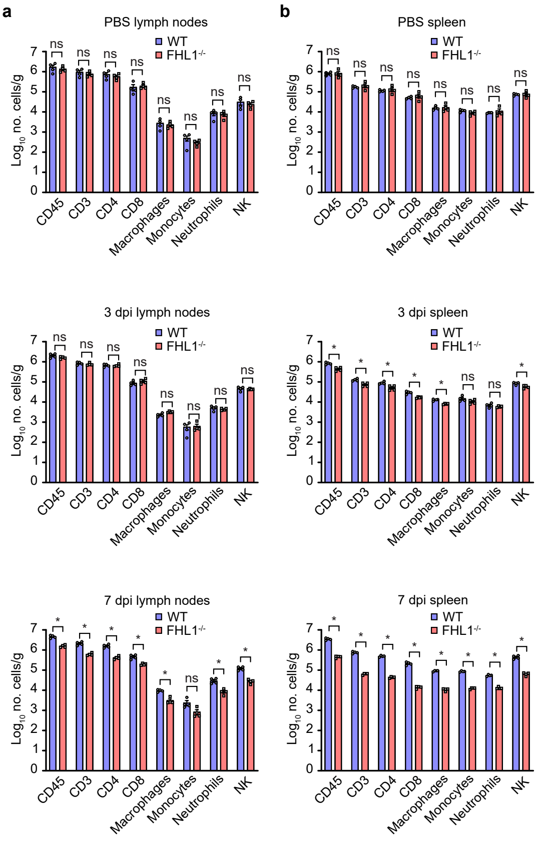

Supplementary Fig S4

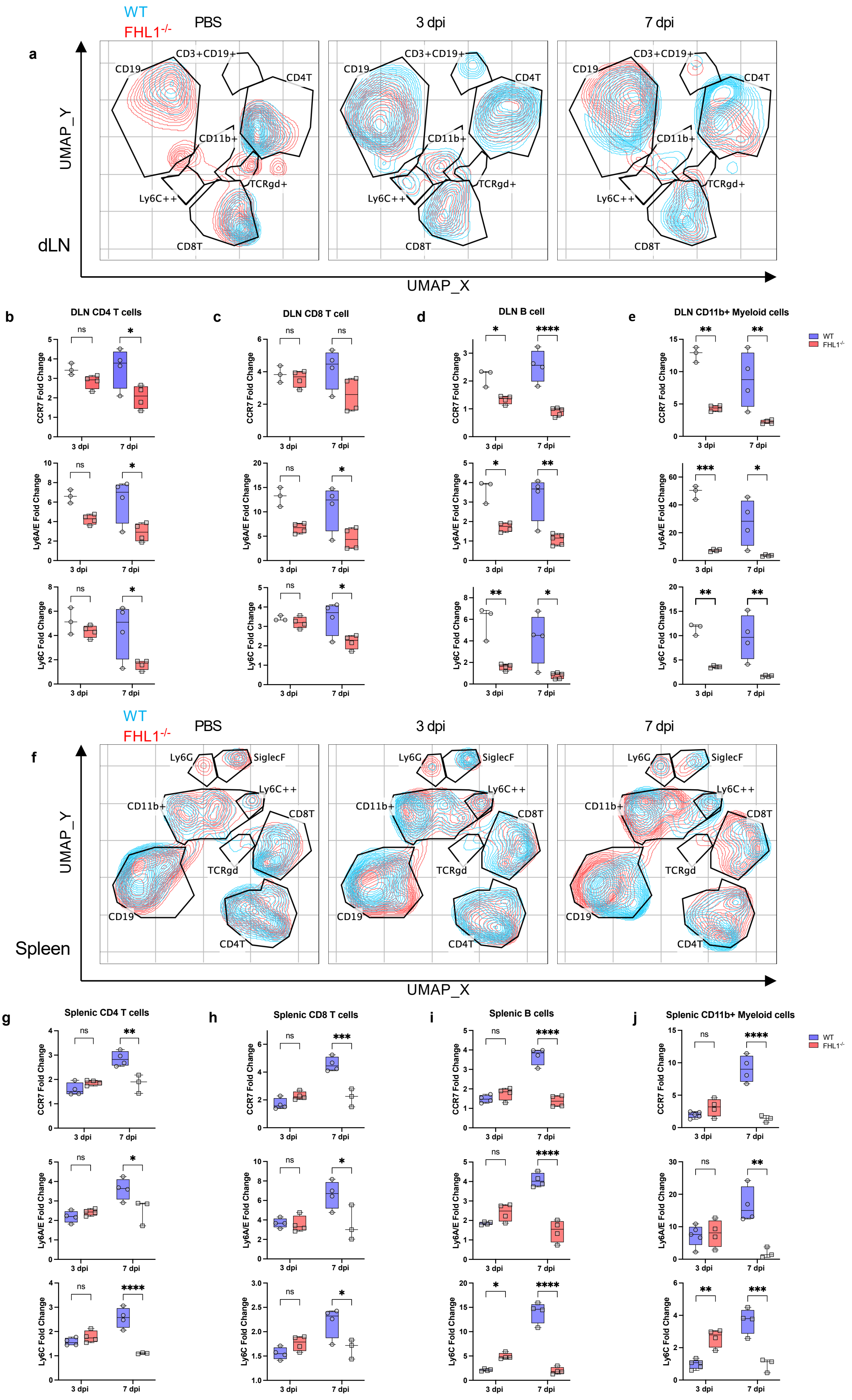

Supplementary Fig S5

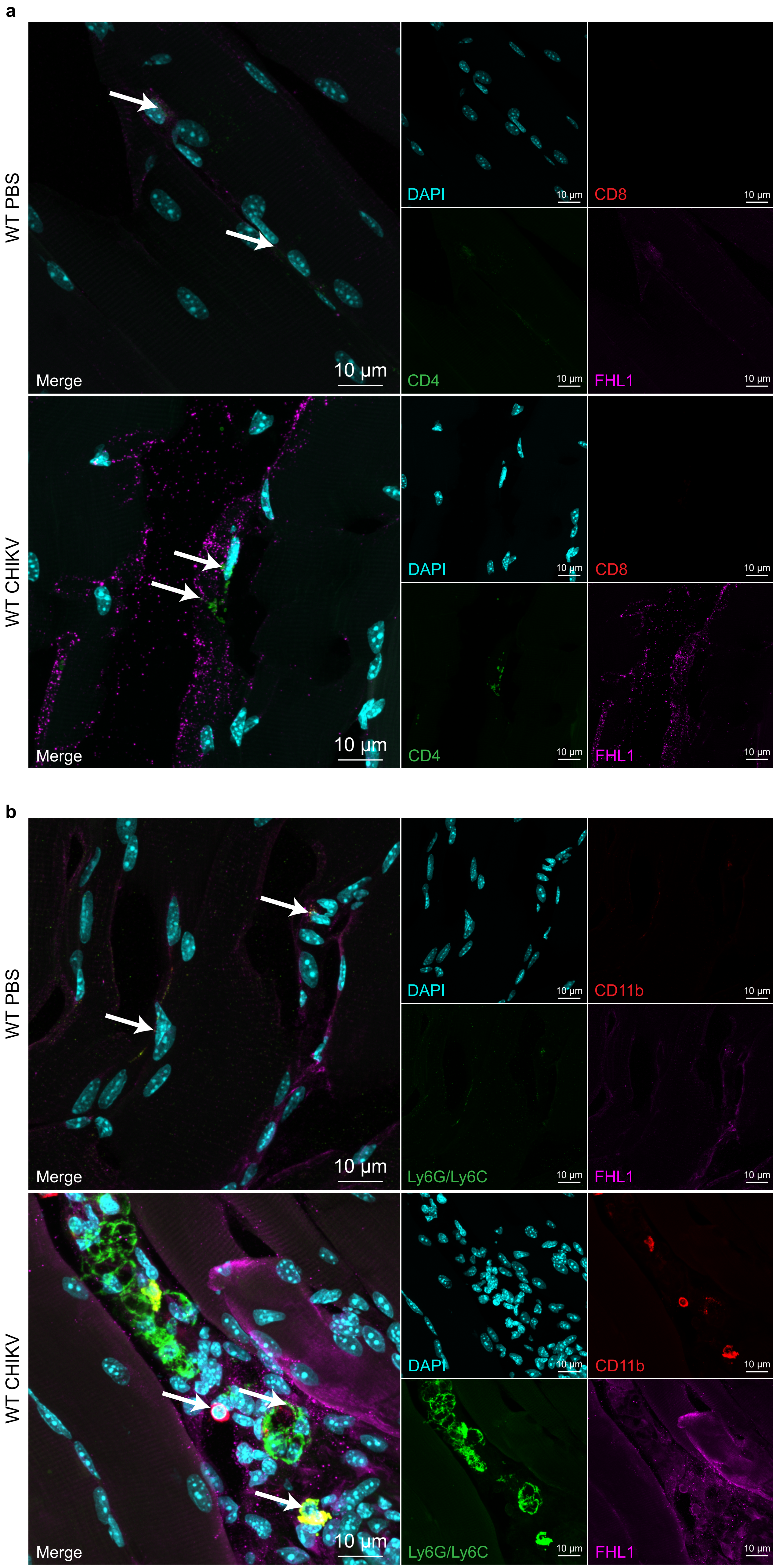

Supplementary Fig S6

a

```
CHIKV-LR2006  1653 SPREYRSSQESAQEASTITSLTHSQFDLSVDGEILPVPSDLDADAPALEPALDDGATHTLPSTTGNLAAVSDWVMSTVPVAPPRRRRGRN
 CHIKV-3del5  1653 SPR--------------------------------------------------------------NLAAVSDWVMSTVPVAPPRRRRGRN
 CHIKV-ΔFHL1  1653 SPREYRSSQESAQEASTITSLTHSQFDLSVDGEILPVPSDLDADAPALEPALDDGATHTLPSTTGNLAAVSDWVMSTVPVAPPRRRRGRN

CHIKV-LR2006  1743 LTVTCDEREGNITPMASVRFFRAELCPVVQETAETRDTAMSLQAPPSTATEPNHPPISFGASSETFPITFGDFNEGEIESLSSELLTFGD
 CHIKV-3del5  1743 LTVTCDEREGNITPMASVRFFRAELCPVVQETAETRDTAMSLQAPPSTATEPNHPPISFGASSETFPITFGDFNEGEIESLSSELLTFGD
 CHIKV-ΔFHL1  1743 LTVTCDEREGNITPMASVRGGRAELCPVVQETAETRDRSMSLGTPPSTATEPNHPPISFGASSETFPITFGDFNEGEIESLSSELLTFGD

CHIKV-LR2006  1833 FLPGEVDDLTDSDWSTCSDTDDELRLDRAGG
 CHIKV-3del5  1833 FLPGEVDDLTDSDWSTCSDTDDELRLDRAGG
 CHIKV-ΔFHL1  1833 FLPGEVDDLTDSDWSTCSDTDDELRLDRAGG
```

b

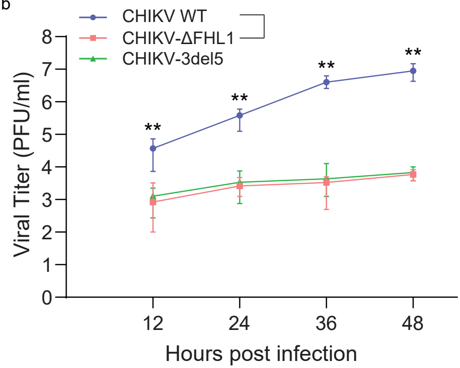

Supplementary Table 1.

	**Healthy control** n = 8	**Acute CHIKV** n = 39	**Chronic CHIKV** n = 17
CHIKV PCR +ve (%)	0 (0/8)	100 (39/39)	41.18 (7/17)
CHIKV IgG +ve (%)	0 (0/8)	100 (5/5)*	70.59 (12/17)
CHIKV IgM +ve (%)	0 (0/8)	50 (1/2)*	11.76 (2/17)
Age (Mean ± S.E.M.)<br><br>(Range)	33.13 ± 2.94<br><br>(22-38)	42.57 ± 2.87<br><br>(11-71)	57.65 ± 3.00<br><br>(29-74)
Gender distribution	3 males<br>5 females	11 males<br>28 females	3 males<br>14 females
Time from disease onset to sample collection (Mean days ± S.E.M.)	N/A	19.13 ± 4.18	258.46 ± 28.78
Fever (%)	N/A	82.05 (32/39)	94.12 (16/17)
Arthralgia (%)	N/A	79.49 (31/39)	100 (17/17)
Rash (%)	N/A	61.54 (24/39)	70.59 (12/17)
Conjunctivitis (%)	N/A	25.64 (10/39)	11.76 (2/17)
Myalgia (%)	N/A	76.92 (30/39)	94.12 (16/17)
Retro-orbital (%)	N/A	48.72 (19/39)	47.06 (8/17)
Lymphadenopathy (%)	N/A	12.82 (5/39)	35.29 (6/17)

*Only available data are presented
N/A : Not applicable

Supplementary Table 2

	Sample collection date	Sample collection location	Donor code	Sample	qPCR ZIKV	qPCR CHIKV	IgM CHIKV	IgG CHIKV	FHL1 Dosage	City (Country NoBrazil)	State	Donor gender M/F	Donor age	Fever	Arthralgia	Rash	Conjunctivitis	Myalgia	Retro-orbital pain	Lymphadenopathy	Duration of symptoms until the date of collection
Healthy control	15/02/2018	University Hospital of Federal University of Sergipe	CTS-2	Serum or Plasma	Negative	Negative	Negative	Negative	1.058321939	Aracaju	Sergipe	F	36	NA	NA	NA	NA	NA	NA	NA	NA
	15/02/2018	University Hospital of Federal University of Sergipe	CTS-12	Serum or Plasma	Negative	Negative	Negative	Negative	0.173431944	Lagarto	Sergipe	M	22	NA	NA	NA	NA	NA	NA	NA	NA
	15/02/2018	University Hospital of Federal University of Sergipe	CTS-14	Serum or Plasma	Negative	Negative	Negative	Negative	0.86971719	Aracaju	Sergipe	F	49	NA	NA	NA	NA	NA	NA	NA	NA
	15/02/2018	University Hospital of Federal University of Sergipe	ECT-5	Serum or Plasma	Negative	Negative	Negative	Negative	1.453283747	Aracaju	Sergipe	F	27	NA	NA	NA	NA	NA	NA	NA	NA
	15/02/2018	University Hospital of Federal University of Sergipe	CTS-9	Serum or Plasma	Negative	Negative	Negative	Negative	0.22719658	Aracaju	Sergipe	M	35	NA	NA	NA	NA	NA	NA	NA	NA
	16/03/2016	University Hospital of Federal University of Sergipe	ARB 525	Serum or Plasma	Negative	Negative	Negative	Negative	5.635624411	Nossa Sra da Glória	Sergipe	F	38	NA	NA	NA	NA	NA	NA	NA	NA
	15/02/2018	University Hospital of Federal University of Sergipe	CTS-17	Serum or Plasma	Negative	Negative	Negative	Negative	0.65930584	Aracaju	Sergipe	F	29	NA	NA	NA	NA	NA	NA	NA	NA
	15/02/2018	University Hospital of Federal University of Sergipe	CTS-6	Serum or Plasma	Negative	Negative	Negative	Negative	0.590762184	Aracaju	Sergipe	M	29	NA	NA	NA	NA	NA	NA	NA	NA
Acute CHIKV	3/03/2016	University Hospital of Federal University of Sergipe	ARB 502	Serum or Plasma	Negative	Positive	NP	NP	8.959865494	Aracaju	Sergipe	F	15	No	No	No	No	No	Yes	No	NS
	9/03/2016	University Hospital of Federal University of Sergipe	ARB 503	Serum or Plasma	Negative	Positive	NP	NP	4.855951183	Aracaju	Sergipe	F	24	No	No	Yes	No	No	No	No	NS
	23/02/2016	University Hospital of Federal University of Sergipe	ARB 506	Serum or Plasma	Negative	Positive	NP	NP	4.791287998	Aracaju	Sergipe	F	NS	No	No	No	No	No	No	No	NS
	7/02/2016	University Hospital of Federal University of Sergipe	ARB 509	Serum or Plasma	Negative	Positive	NP	NP	3.766086413	Aracaju	Sergipe	F	40	No	No	No	No	No	No	No	NS
	22/03/2016	University Hospital of Federal University of Sergipe	ARB 511	Serum or Plasma	Negative	Positive	NP	NP	2.654932248	Aracaju	Sergipe	F	40	No	No	No	No	No	No	No	NS
	28/03/2016	University Hospital of Federal University of Sergipe	ARB 532	Serum or Plasma	Negative	Positive	NP	NP	24.02789564	Aracaju	Sergipe	M	50	Yes	Yes	Yes	Yes	Yes	Yes	No	NS
	23/03/2016	University Hospital of Federal University of Sergipe	ARB 671	Serum or Plasma	Negative	Positive	NP	NP	5.635624411	Aracaju	Sergipe	F	41	Yes	Yes	Yes	No	Yes	No	No	NS
	28/03/2016	University Hospital of Federal University of Sergipe	ARB 534	Serum or Plasma	Negative	Positive	NP	NP	6.046894427	Aracaju	Sergipe	F	11	Yes	Yes	Yes	Yes	Yes	Yes	No	NS
	28/03/2016	University Hospital of Federal University of Sergipe	ARB 551	Serum or Plasma	Negative	Positive	NP	NP	20.83195866	Itabaiana	Sergipe	F	30	Yes	Yes	No	Yes	Yes	Yes	No	1
	30/03/2016	University Hospital of Federal University of Sergipe	ARB 560	Serum or Plasma	Negative	Positive	NP	NP	5.624108132	Itabaiana	Sergipe	F	24	Yes	No	No	No	Yes	Yes	No	2
	1/04/2016	University Hospital of Federal University of Sergipe	ARB 561	Serum or Plasma	Negative	Positive	NP	NP	28.74728703	Aracaju	Sergipe	M	57	Yes	Yes	No	No	No	No	No	NS
	1/04/2016	University Hospital of Federal University of Sergipe	ARB 562	Serum or Plasma	Negative	Positive	NP	NP	4.100931968	Macambira	Sergipe	F	58	Yes	Yes	Yes	No	Yes	Yes	No	4
	4/04/2016	University Hospital of Federal University of Sergipe	ARB 578	Serum or Plasma	Negative	Positive	NP	NP	19.38155156	Macambira	Sergipe	M	45	Yes	Yes	Yes	No	Yes	No	No	1
	14/04/2016	University Hospital of Federal University of Sergipe	ARB 586	Serum or Plasma	Negative	Positive	NP	NP	4.511580301	Campo do Brito	Sergipe	F	25	Yes	Yes	Yes	No	Yes	No	No	15
	4/04/2016	University Hospital of Federal University of Sergipe	ARB 595	Serum or Plasma	Negative	Positive	NP	NP	38.72402076	Macambira	Sergipe	F	26	Yes	Yes	No	Yes	Yes	Yes	No	2
	2/06/2016	University Hospital of Federal University of Sergipe	ARB 601	Serum or Plasma	Positive	Positive	Negative	Positive	2.619440202	Campo do Brito	Sergipe	M	37	Yes	Yes	No	No	Yes	No	No	2
	25/05/2016	University Hospital of Federal University of Sergipe	ARB 604	Serum or Plasma	Negative	Positive	NP	NP	20.8788219	Aracaju	Sergipe	F	36	Yes	Yes	Yes	No	Yes	No	No	NS
	14/04/2016	University Hospital of Federal University of Sergipe	ARB 633	Serum or Plasma	Negative	Positive	NP	NP	3.656506821	Campo do Brito	Sergipe	F	71	Yes	Yes	No	Yes	Yes	Yes	Yes	60
	14/04/2016	University Hospital of Federal University of Sergipe	ARB 637	Serum or Plasma	Negative	Positive	NP	NP	5.178743732	Campo do Brito	Sergipe	F	50	Yes	Yes	Yes	No	No	No	No	5
	10/06/2016	University Hospital of Federal University of Sergipe	ARB 645	Serum or Plasma	Negative	Positive	NP	NP	3.327423889	Aracaju	Sergipe	F	NS	Yes	Yes	Yes	No	Yes	No	No	NS
	1/07/2016	University Hospital of Federal University of Sergipe	ARB 656	Serum or Plasma	Negative	Positive	NP	NP	8.778701473	Aracaju	Sergipe	F	31	Yes	Yes	Yes	Yes	No	Yes	Yes	NS
	1/07/2016	University Hospital of Federal University of Sergipe	ARB 659	Serum or Plasma	Negative	Positive	NP	NP	7.57046375	Aracaju	Sergipe	F	63	Yes	Yes	Yes	No	Yes	No	No	7
	20/07/2016	University Hospital of Federal University of Sergipe	ARB 670	Serum or Plasma	Negative	Positive	NP	NP	4.144375028	Aracaju	Sergipe	F	33	Yes	No	Yes	No	Yes	Yes	No	NS
	1/08/2016	University Hospital of Federal University of Sergipe	ARB 675	Serum or Plasma	Negative	Positive	NP	NP	3.19592218	Aracaju	Sergipe	F	65	Yes	Yes	Yes	No	Yes	No	No	60
	5/08/2016	University Hospital of Federal University of Sergipe	ARB 678	Serum or Plasma	Negative	Positive	NP	Positive	2.780376104	Nossa Sra do Socorro	Sergipe	M	52	Yes	Yes	Yes	No	Yes	Yes	No	>30
	2/04/2016	University Hospital of Federal University of Sergipe	ARB 680	Serum or Plasma	Negative	Positive	NP	NP	9.082947826	Moita Bonita	Sergipe	M	19	Yes	Yes	No	Yes	Yes	Yes	No	1
	28/04/2016	University Hospital of Federal University of Sergipe	ARB 706	Serum or Plasma	Negative	Positive	NP	NP	3.292296254	Campo do Brito	Sergipe	F	31	Yes	Yes	Yes	Yes	Yes	Yes	Yes	30
	16/04/2016	University Hospital of Federal University of Sergipe	ARB 756	Serum or Plasma	Negative	Positive	NP	NP	7.792391538	Itabaianinha	Sergipe	F	75	Yes	Yes	Yes	Yes	Yes	No	No	15
	16/04/2016	University Hospital of Federal University of Sergipe	ARB 764	Serum or Plasma	Negative	Positive	NP	NP	5.934222111	Itabaianinha	Sergipe	F	68	Yes	Yes	Yes	No	Yes	No	No	NS
	16/07/2016	University Hospital of Federal University of Sergipe	ARB 788	Serum or Plasma	Negative	Positive	NP	NP	5.49953521	Itabaianinha	Sergipe	M	37	Yes	Yes	No	No	Yes	Yes	No	30
	16/07/2016	University Hospital of Federal University of Sergipe	ARB 792	Serum or Plasma	Negative	Positive	NP	NP	6.199598251	Itabaianinha	Sergipe	M	65	Yes	Yes	Yes	No	Yes	Yes	No	15
	12/08/2016	University Hospital of Federal University of Sergipe	ARB 810	Serum or Plasma	Negative	Positive	NP	Positive	6.526551938	Nossa Sra do Socorro	Sergipe	F	51	Yes	Yes	Yes	No	Yes	Yes	Yes	22
	18/08/2016	University Hospital of Federal University of Sergipe	ARB 811	Serum or Plasma	Negative	Positive	NP	NP	9.04223194	Campo do Brito	Sergipe	M	41	Yes	No	No	No	Yes	Yes	No	15
	18/08/2016	University Hospital of Federal University of Sergipe	ARB 812	Serum or Plasma	Negative	Positive	NP	NP	20.65637467	Campo do Brito	Sergipe	F	38	Yes	Yes	Yes	No	Yes	Yes	No	15
	8/09/2016	University Hospital of Federal University of Sergipe	ARB 820	Serum or Plasma	Negative	Positive	NP	NP	13.00538489	Aracaju	Sergipe	M	12	Yes	Yes	Yes	No	Yes	Yes	Yes	3
	2/09/2016	University Hospital of Federal University of Sergipe	ARB 821	Serum or Plasma	Negative	Positive	NP	NP	6.059587526	Malhador	Sergipe	F	34	No	Yes	Yes	No	Yes	No	No	60
	15/09/2017	University Hospital of Federal University of Sergipe	ARB 901	Serum or Plasma	Negative	Positive (CSF)	Borderline	Positive	2.412619153	Aracaju	Sergipe	M	68	No	Yes	No	No	No	No	No	NS
	16/04/2016	University Hospital of Federal University of Sergipe	ARB 725	Serum or Plasma	Negative	Positive	NP	NP	4.754912575	Itabaiana	Sergipe	F	61	Yes	Yes	No	Yes	Yes	No	No	45
	12/08/2016	University Hospital of Federal University of Sergipe	ARB 809	Serum or Plasma	Positive	Positive	NP	Positive	4.405045698	Aracaju	Sergipe	F	51	Yes	Yes	Yes	No	Yes	No	No	30
Chronic CHIKV	16/04/2016	University Hospital of Federal University of Sergipe	ARB 735	Serum or Plasma	Negative	Positive	NP	NP	1.378375735	Itabaianinha	Sergipe	F	74	Yes	Yes	No	No	Yes	No	No	120
	14/05/2016	University Hospital of Federal University of Sergipe	ARB 783	Serum or Plasma	Negative	Positive	NP	NP	13.81044377	Itabaianinha	Sergipe	F	58	Yes	Yes	Yes	No	Yes	Yes	Yes	180
	8/08/2016	University Hospital of Federal University of Sergipe	ARB 802	Serum or Plasma	Negative	Positive	NP	Positive	0.889025785	Aracaju	Sergipe	F	40	Yes	Yes	Yes	No	Yes	No	No	270
	12/08/2016	University Hospital of Federal University of Sergipe	ARB 805	Serum or Plasma	Negative	Positive	NP	Positive	7.723299436	Laranjeiras	Sergipe	F	57	Yes	Yes	Yes	Yes	Yes	Yes	No	240
	19/08/2016	University Hospital of Federal University of Sergipe	ARB 814	Serum or Plasma	Negative	Positive	NP	Positive	5.77635839	Aracaju	Sergipe	F	29	Yes	Yes	Yes	No	Yes	No	No	210
	4/11/2016	University Hospital of Federal University of Sergipe	ARB 845	Serum or Plasma	Negative	Negative	NP	Positive	7.307538957	Aracaju	Sergipe	M	68	Yes	Yes	Yes	No	Yes	Yes	Yes	180
	25/11/2016	University Hospital of Federal University of Sergipe	ARB 862	Serum or Plasma	Negative	Positive	NP	Positive	5.125546655	Aracaju	Sergipe	F	73	Yes	Yes	Yes	No	Yes	Yes	Yes	120
	30/11/2016	University Hospital of Federal University of Sergipe	ARB 864	Serum or Plasma	Negative	Negative	NP	Positive	4.174650538	Aracaju	Sergipe	M	63	No	Yes	Yes	No	Yes	Yes	No	360
	9/12/2016	University Hospital of Federal University of Sergipe	ARB 870	Serum or Plasma	Negative	Negative	NP	Positive	2.860195547	Boquim	Sergipe	F	43	Yes	Yes	Yes	No	Yes	Yes	Yes	240
	17/01/2017	University Hospital of Federal University of Sergipe	ARB 875	Serum or Plasma	Negative	Negative	NP	Positive	4.141659554	Capela	Sergipe	M	47	Yes	Yes	Yes	No	Yes	Yes	No	380
	10/02/2017	University Hospital of Federal University of Sergipe	ARB 885	Serum or Plasma	Negative	Negative	NP	Positive	2.029081322	Aracaju	Sergipe	F	48	Yes	Yes	Yes	Yes	Yes	Yes	No	365
	21/02/2017	University Hospital of Federal University of Sergipe	ARB 889	Serum or Plasma	Negative	Negative	NP	Positive	2.043316776	Aracaju	Sergipe	F	62	Yes	Yes	Yes	No	Yes	No	Yes	455
	20/06/2017	University Hospital of Federal University of Sergipe	ARB 897	Serum or Plasma	Negative	Negative	Positive	NP	0.648189983	Aracaju	Sergipe	F	65	Yes	Yes	Yes	No	No	No	Yes	>90
	4/09/2017	University Hospital of Federal University of Sergipe	ARB 899	Serum or Plasma	Positive	Positive (CSF)	Negative	NP	3.548850106	Itabaiana	Sergipe	F	60	Yes	Yes	No	No	Yes	No	No	240
	18/09/2017	University Hospital of Federal University of Sergipe	ARB 902	Serum or Plasma	Negative	Negative	NP	Positive	4.256274731	Itabaiana	Sergipe	F	60	Yes	Yes	No	No	Yes	No	No	NS
	4/09/2019	University Hospital of Federal University of Sergipe	ARB 999	Serum or Plasma	Negative	Negative	NP	Positive	4.976779776	NS	Sergipe	F	69	Yes	Yes	No	No	Yes	No	No	>90
	17/10/2019	University Hospital of Federal University of Sergipe	ARB 998	Serum or Plasma	Negative	Negative	Positive	Negative	0.633030291	NS	Sergipe	F	64	Yes	Yes	No	No	Yes	No	No	>90

NP: not performed
NS: not specified
NA: not applicable
No: lack of this symptom
Yes: presence of this symptom

Supplementary Table 3

Project	Infection cohort	Subject Code	Gender	Age (Range)	CollectionDate	CollectionTimePoint	Days since symptom onset	Fever	Rash	Malaise	Lymphad	Headaches	Arthralgia	Myalgia activity	Body pain	Longer sleep	Post activity tired	Poor sleep	Tired activity
N/A	Control	A540022731959007	male	41-50	24/05/2022	N/A	N/A	N/A	N/A	N/A	N/A	N/A	N/A	N/A	N/A	N/A	N/A	N/A	N/A
N/A	Control	A540022743120000	female	21-30	25/05/2022	N/A	N/A	N/A	N/A	N/A	N/A	N/A	N/A	N/A	N/A	N/A	N/A	N/A	N/A
N/A	Control	A54002274310700V	male	71-80	26/05/2022	N/A	N/A	N/A	N/A	N/A	N/A	N/A	N/A	N/A	N/A	N/A	N/A	N/A	N/A
N/A	Control	A540022743101006	male	51-60	27/05/2022	N/A	N/A	N/A	N/A	N/A	N/A	N/A	N/A	N/A	N/A	N/A	N/A	N/A	N/A
N/A	Control	A54002284381900E	female	21-30	28/05/2022	N/A	N/A	N/A	N/A	N/A	N/A	N/A	N/A	N/A	N/A	N/A	N/A	N/A	N/A
N/A	Control	A54002284380000*	male	31-40	29/05/2022	N/A	N/A	N/A	N/A	N/A	N/A	N/A	N/A	N/A	N/A	N/A	N/A	N/A	N/A
N/A	Control	A540022835864004	male	21-30	30/05/2022	N/A	N/A	N/A	N/A	N/A	N/A	N/A	N/A	N/A	N/A	N/A	N/A	N/A	N/A
N/A	Control	A540022843782008	female	41-50	31/05/2022	N/A	N/A	N/A	N/A	N/A	N/A	N/A	N/A	N/A	N/A	N/A	N/A	N/A	N/A
N/A	Control	A54002284379900R	male	71-80	1/06/2022	N/A	N/A	N/A	N/A	N/A	N/A	N/A	N/A	N/A	N/A	N/A	N/A	N/A	N/A
N/A	Control	A54002284165300L	female	61-70	2/06/2022	N/A	N/A	N/A	N/A	N/A	N/A	N/A	N/A	N/A	N/A	N/A	N/A	N/A	N/A
DIOS	RRV	1594	female	51-60	31/05/2007	Baseline	17	0	0	1	0	0	1	2	1	2	1	0	2
DIOS	RRV	1627	male	41-50	5/03/2008	Baseline	21	0	0	0	0	0	2	2	2	0	1	2	2
DIOS	RRV	1630	male	31-40	19/03/2008	Baseline	20	0	2	0	1	0	2	2	2	2	2	2	2
DIOS	RRV	2021	male	21-30	20/05/1997	Baseline	20	0	0	0	0	0	0	0	0	0	0	0	0
DIOS	RRV	2085	male	21-30	16/12/1998	Baseline	19	1	0	1	0	0	2	1	2	1	1	1	1
DIOS	RRV	2145	male	41-50	16/02/2000	Baseline	21	0	0	2	0	0	2	0	0	0	1	0	1
DIOS	RRV	2156	female	41-50	2/05/2000	Baseline	17	0	0	0	0	0	2	2	2	1	1	2	0
DIOS	RRV	2164	male	41-50	11/01/2001	Baseline	22	0	0	1	1	0	1	0	2	1	0	1	1
DIOS	RRV	3012	female	51-60	15/11/2001	Baseline	16	1	0	1	1	1	2	1	1	1	1	2	2
DIOS	RRV	3015	female	11-20	12/12/2001	Baseline	19	2	1	1	0	1	0	2	1	2	2	2	2

N/A = Not applicable

Actual questions

Baseline - Fevers?
Baseline - had a rash
Baseline - generally felt unwell
Baseline - had tender glands in your neck or else where
Baseline - Headaches?
Baseline - Joint pain?
Baseline - Muscle pain after activity?
Baseline - Pains in your arms or legs?
Baseline - Needing to sleep longer?
Baseline - Prolonged tiredness after activity?
Baseline - Poor sleep?
Baseline - Tired muscles after activity?

Response code: since the illness onset?

0 None of the time or some of the time
1 A good part of the time
2 Most of the time or all of the time

Supplementary Table 4. Antibody labels for mass cytometry.

Specificity	Clone #	Conjugated Mass Label
**CD16/32**	2.4G2	Unlabelled (Fc Block)
**B220**	RA3-6B2	89Y-B220
**CD45**	30-F11	104Pd-CD45
**CD45**	30-F11	106Pd-CD45
**CD45**	30-F11	108Pd-CD45
**CD45**	30-F11	110Pd-CD45
**CD69**	H1.2F3	139La-CD69
**Ly6G**	1A8	141Pr-Ly6G
**CD11c**	N418	142Nd-CD11c
**CD194**	2G12	143Nd-CD194
**Siglec-F**	E50-2440	146Nd-Siglec-F
**CD11b**	M1/70	148Nd-CD11b
**CD80**	16-10A1	149Sm-CD80
**CD103**	2E7	150Nd-CD103
**F4/80**	BM8	151Eu-F4/80
**CD4**	RM4-5	153Eu-CD4
**CD163**	S15049I	154Gd-CD163
**CD138**	281-2	155Gd-CD138
**CD48**	HM48-1	156Gd-CD48
**FOXP3**	FJK-16s	158Gd-FoxP3
**CD117**	2B8	159Tb-CD117
**CD62L**	MEL-14	160Gd-CD62L
**CD25**	3C7	161Dy-CD25
**Ki67**	11F6	162Er-Ki67
**CD197**	4B12	163Dy-CD197
**Ly6A/E**	D7	164Er-Ly6CA/E
**CD115**	AFS98	165Ho-CD115
**CD19**	6D5	166Er-CD19
**CD185**	2G8	167Er-CD185
**CD8a**	53-6.7	168Er-CD8a
**TCRγδ**	GL3	169Tm-TCRgd
**NK1.1**	PK136 (B6)	170Er-NK1.1
**CD44**	IM7	171Yb-CD44
**CD127**	A7R34	173Yb-CD127
**MHC-II IA/IE**	M5/114.15.2	174Yb-MHCII-IA-IE
**CD192**	475301	175Lu-CCR2
**Ly6C**	HK1.4	176Lu-Ly6C
**DNA label**	N/A	191/193Ir
**Cisplatin (Live/dead)**	N/A	194/195Pt
**CD3ε**	145-2C11	209Bi-CD3

Supplementary information_Gating strategy

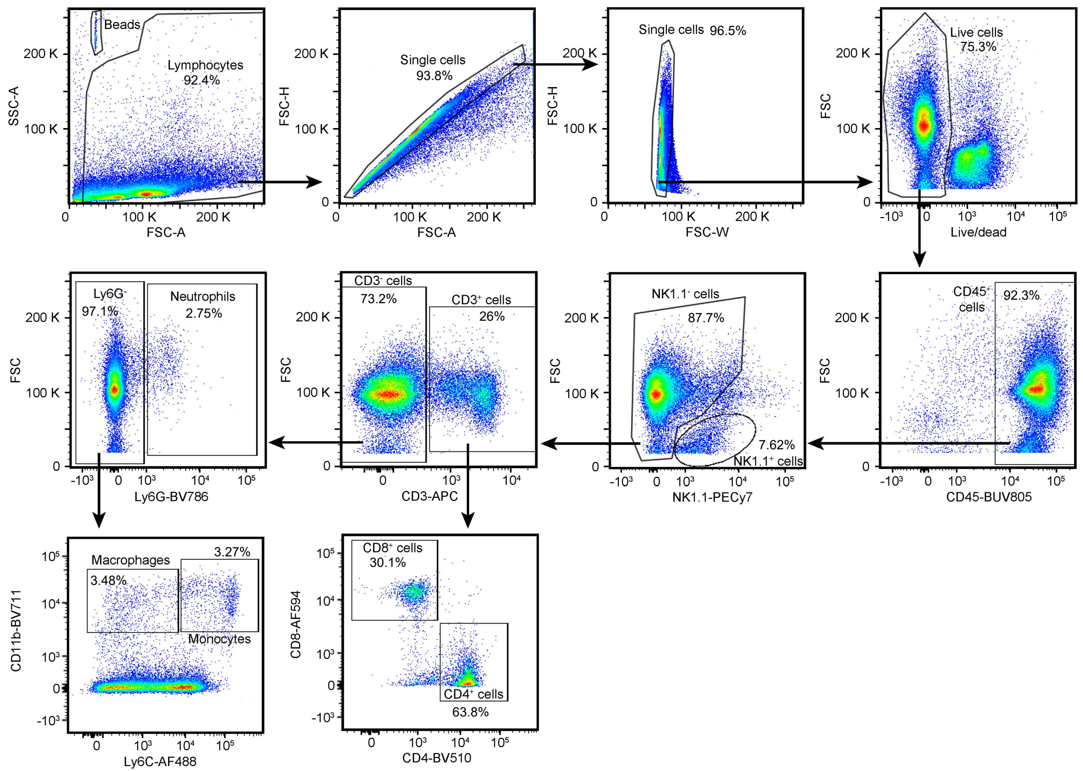

Supplement: Supplementary file 1 — Supplementary Information [file 41467_2023_42330_MOESM1_ESM.pdf]
