# Supplementary material for: FHL1 promotes chikungunya and o’nyong-nyong virus infection and pathogenesis with implications for alphavirus vaccine design
Source: Nat Commun. 2023 Oct 26;14:6605. doi: 10.1038/s41467-023-42330-2 (PMC10603155; doi:10.1038/s41467-023-42330-2)
Supplement: Supplementary file 4 — Supplementary Figure legend [file 41467_2023_42330_MOESM4_ESM.docx]

**Supplementary** **Fig. 1. Generation of FHL1-knock-out mice.** FHL1^-/-^ mice were made by injecting 20 ng/μL Cas9 mRNA and 10 ng/μL each sgRNAs into the cytoplasm of fertilized one-cell-stage embryos generated from WT C57BL/6J breeders. Twenty-four hours later, two-cell-stage embryos were transferred into the uterus of pseudopregnant mice. Viable offspring were genotyped using PCR and analyzed using next-generation sequencing (a). The genotype of F_0_ mosaic offspring were analyzed by PCR. Agarose gel with representative PCR products showing the FHL1 genotyping. The expected bands were 387 bp for the deleted allele and 309 bp for the WT allele (b). The F_0_ mosaic offspring of #16, #17, #19 and #23 were selected for next-generation sequencing. InDel mutations are highlighted in yellow (c). InDel mutation of #17, #19 and #23 (d). Establishment of germline homozygosity. FHL1 mosaic mice were back-crossed with WT mice to generate heterozygous FHL1^+/-^ mice. FHL1^+/-^ mice were then in-bred to generate FHL1^-/-^ mice. All breeders/litters were genotyped by PCR to ensure FHL1 was knocked out prior to being paired for mating and/or used for experiments (e). Quadriceps, heart and tail samples obtained from WT or FHL1^-/-^ mice were analyzed by Western Blot for the expression of FHL1 (f). Source data are provided as a Source Data file.

**Supplementary** **Fig. 2. FHL1 is associated with articular cartilage damage in the ankles of mice with CHIKV disease**. WT and FHL1^-/-^ mice were infected with CHIKV at 10^4^ PFU or mock-infected with PBS. The ipsilateral ankles were collected at 3 and 7 dpi and processed for safranin-O staining (a, c, e). The microscopy images shown are representative of n = 10 mice per group. The thickness of the articular cartilage was analyzed using ImageScope (yellow lines). Dots represent individual animals (n = 5); the data shown are representative of two independent experiments (b, d, f). Data are presented as box and whisker ± SD with the mean indicated by a line across the box, maximum to minimum points (ns, nonsignificant; **, P < 0.01; Mann-Whitney test). Source data are provided as a Source Data file.

**Supplementary** **Fig. 3. FHL1 is associated with reduced immune cell populations in the spleen and lymph nodes of mice with CHIKV disease.** WT and FHL1^-/-^ mice were infected with 10^4^ PFU CHIKV or mock-infected with PBS. The DLNs (a) and spleen (b) were harvested at 3 and 7 dpi and processed for flow cytometry. Cells were analyzed to determine the numbers of leukocytes (CD45^+^), CD4+ (CD3^+^, CD4^+^) and CD8+ (CD3^+^, CD8^+^) T cells, macrophages (CD11b^+^, Ly6G^-^, and Ly6C^-^), monocytes (CD11b^+^, Ly6G^-^, and Ly6C^+^), neutrophils (CD3^-^, Ly6G^+^) and NK cells (NK1.1^+^). Dots represent individual animals (n = 5). Data are presented as the mean ± SEM (ns, nonsignificant; *, P < 0.05; Mann-Whitney test). Source data are provided as a Source Data file. Gating strategy is attached in Supplementary Information file.

**Supplementary Fig. 4. FHL1 is associated with reduced cell immune phenotypes.** WT and FHL1^-/-^ mice were infected with 10^4^ PFU CHIKV. The DLNs and spleen were harvested at 3 and 7 dpi and processed for mass cytometry. Position shifts on the UMAP contour plots for DLNs (a) and spleen (f) as a result of cell phenotype change. Clusters were identified and gated based on the expression of traditional immune cell markers, including CD3, CD4, CD8, CD19, CD11b, Ly6C, Ly6G, SiglecF and TCRγδ. CCR7, Ly6A/E and Ly6C were identified as major contributors to cell phenotype shifts and fold-changes of these markers were plotted for CD4 T cells (b), CD8 T cells (c), B cells (d) and CD11b^+^ Myeloid cells (e) in the DLNs. Fold changes were also plotted for splenic CD4 T cells (g), CD8 T cells (h), B cells (i) and CD11b^+^ Myeloid cells (j). Dots represent individual animals; n = 3-5 mice per group. Data are presented as box and whisker ± SD with the mean indicated by a line across the box, maximum to minimum points (ns, nonsignificant; *, P < 0.05; **, P<0.01; ***, P<0.001; ****, P<0.0001; two-way ANOVA, Tukey’s comparison test). Source data are provided as a Source Data file.

**Supplementary** **Fig. 5. Immunofluorescence analyses of FHL1 co-expression in immune cells in mice quadriceps.** Immunofluorescence analysis of quadriceps from mock or CHIKV-infected WT mice (mock, n = 5; CHIKV, n = 8) at 7 dpi (a-b). Cryosections were labelled for CD4 and CD8 (T cells), FHL1 and DAPI (a), and Ly6G/Ly6C (neutrophils/monocytes), CD11b (macrophages), FHL1, and DAPI (nucleus) (b). The co-expression of FHL1 in different immune cells are shown in the images. The confocal microscopy images were acquired using a 40× (with 2× zoom) objective. Scale bars in panel = 10 µm for 40× (with 2× zoom) objective.

**Supplementary** **Fig. 6. FHL1 is required for optimal infection of CHIKV in vitro.** Amino acid sequence alignment for nsP3 HVD between CHIKV-LR2006 (GenBank: [EU224268.1](https://www.ncbi.nlm.nih.gov/nuccore/EU224268.1)), CHIKV-3del5 and CHIKV-ΔFHL1. The six mutations in CHIKV-ΔFHL1 are highlighted in yellow color. The red box indicates FHL1 interaction region (a). Multi-step growth curves of CHIKV, CHIKV-ΔFHL1 and CHIKV-3del5 (b). Vero cells were infected at an MOI of 0.01. Data are presented as the mean and error from two experiments each performed in five technical replicates (**, P < 0.05; Mann-Whitney test). Source data are provided as a Source Data file.

**Supplementary Table. 1. Chikungunya patient information summary.**

**Supplementary Table. 2. Demographics, clinical symptoms, and duration of clinical symptoms until days of collection presented in chikungunya patients.**

**Supplementary Table. 3. Demographics, days since symptoms onset and response code of clinical symptoms presented in RRV disease patients.**

**Supplementary Table. 4. Antibody labels for mass cytometry.**

**Supplementary Information. The gating strategy.**
